# The Diseases of Females: Including Those of Pregnancy and Childbed

**Published:** 1843-04

**Authors:** 


Art. III.—*The Diseases of Females: including those of Pregnancy and Childbed.*—By Fleetwood Churchill ,M. D., author of the "Theory and Practice of Midwifery;" Licentate of the King and Queen's College of Physicians in Ireland, &c. &c. Second American edition. *With notes*, by Robert M. Huston, M. D., Professor of Materia Medica and General Therapeutics, &c. &c. 1 vol., 8vo., pp. 575. Philadelphia, Lea & Blanchard, 1843.

The two parts of which this volume is composed were originally published separately, that "On the Diseases of Females" first, followed after an interval by the "Diseases incident to Pregnancy and Childbed." They are very properly combined in the present edition, and constitute by far the most complete and systematic work on the subject that we know. Both are compilations for the most part, and do not claim to be any thing more. The author has intended to make the work acceptable both to the junior and senior student; to this end, to use his own language, "it has been arranged, in the present volume, that the *text* shall contain an ample outline of the history, pathology, symptoms and treatment of the diseases, without any detail of controversies or conflicting opinions, which are given in full in the *notes* appended to each page; so that the junior student, by confining his attention to the text, may acquire elementary information, which may be subsequently extended by consulting the notes and references."

The notes of the American editor are not very numerous, but pertinent, and we would add *judicious*, if we had not such a horror of the word. C.

